# Release of Endocannabinoids into the Cerebrospinal Fluid during the Induction of the Trigemino-Hypoglossal Reflex in Rats

**DOI:** 10.3390/cimb44050164

**Published:** 2022-05-23

**Authors:** Marek Zubrzycki, Maria Zubrzycka, Grzegorz Wysiadecki, Janusz Szemraj, Hanna Jerczynska, Mariusz Stasiolek

**Affiliations:** 1 Department of Cardiac Surgery and Transplantology, The Cardinal Stefan Wyszynski Institute of Cardiology, Alpejska 42, 04-628 Warsaw, Poland; 2 Department of Clinical Physiology, Faculty of Medicine, Medical University of Lodz, Mazowiecka 6/8, 92-215 Lodz, Poland; maria.pawelska-zubrzycka@umed.lodz.pl; 3 Department of Normal and Clinical Anatomy, Chair of Anatomy and Histology, Medical University of Lodz, Żeligowskiego 7/9, 90-752 Lodz, Poland; grzegorz.wysiadecki@umed.lodz.pl; 4 Department of Medical Biochemistry, Medical University of Lodz, Mazowiecka 6/8, 92-215 Lodz, Poland; janusz.szemraj@umed.lodz.pl; 5 Central Scientific Laboratory (CoreLab), Medical University of Lodz, Mazowiecka 6/8, 92-215 Lodz, Poland; hanna.jerczynska@umed.lodz.pl; 6 Department of Neurology, Medical University of Lodz, Kopcinskiego 22, 90-153 Lodz, Poland; mariusz.stasiolek@umed.lodz.pl

**Keywords:** cerebrospinal fluid, orofacial pain, endocannabinoids, cannabinoid receptors, URB597

## Abstract

The endocannabinoid system (ECS) plays an important role in pain processing and modulation. Since the specific effects of endocannabinoids within the orofacial area are largely unknown, we aimed to determine whether an increase in the endocannabinoid concentration in the cerebrospinal fluid (CSF) caused by the peripheral administration of the FAAH inhibitor URB597 and tooth pulp stimulation would affect the transmission of impulses between the sensory and motor centers localized in the vicinity of the third and fourth cerebral ventricles. The study objectives were evaluated on rats using a method that allowed the recording of the amplitude of evoked tongue jerks (ETJ) in response to noxious tooth pulp stimulation and URB597 treatment. The amplitude of ETJ was a measure of the effect of endocannabinoids on the neural structures. The concentrations of the endocannabinoids tested (AEA and 2-AG) were determined in the CSF, along with the expression of the cannabinoid receptors (CB1 and CB2) in the tissues of the mesencephalon, thalamus, and hypothalamus. We demonstrated that anandamide (AEA), but not 2-arachidonoylglycerol (2-AG), was significantly increased in the CSF after treatment with a FAAH inhibitor, while tooth pulp stimulation had no effect on the AEA and 2-AG concentrations in the CSF. We also found positive correlations between the CSF AEA concentration and cannabinoid receptor type 1 (CB1R) expression in the brain, and between 2-AG and cannabinoid receptor type 2 (CB2R), and negative correlations between the CSF concentration of AEA and brain CB2R expression, and between 2-AG and CB1R. Our study shows that endogenous AEA, which diffuses through the cerebroventricular ependyma into CSF and exerts a modulatory effect mediated by CB1Rs, alters the properties of neurons in the trigeminal sensory nuclei, interneurons, and motoneurons of the hypoglossal nerve. In addition, our findings may be consistent with the emerging concept that AEA and 2-AG have different regulatory mechanisms because they are involved differently in orofacial pain. We also suggest that FAAH inhibition may offer a therapeutic approach to the treatment of orofacial pain.

## 1. Introduction

Orofacial pain is defined as pain caused by a dysfunction or a disease of the somatosensory nervous system [1]. It has a significant impact on everyday activity, functioning, and quality of life in humans. Despite the progress in pain research, there is still no effective treatment that would ensure the complete elimination of this kind of pain. Therefore, new treatment options have been constantly sought. The results of preclinical research suggest that endocannabinoids may become new molecular targets in the development of medications for headaches, including orofacial pain [2,3,4,5].

The endocannabinoid system (ECS) consists of “classic” type 1 cannabinoid receptors (CB1R) and type 2 cannabinoid receptors (CB2R), as well as the endogenous ligands anandamide (AEA) and 2-arachidonoyl glycerol (2-AG), and enzymes that synthesize and degrade these compounds [4,6,7]. The inactivation of AEA proceeds primarily via the fatty acid amide hydrolase (FAAH) enzyme, expressed on post-synaptic somata and dendrites, whereas the inactivation of 2-AG occurs predominantly through monoacylglycerol lipase (MAGL), located on presynaptic axon terminals [6,8]. The enzymes responsible for the degradation pathways are critical in the modulation of the AEA and 2-AG levels [2]. The action of FAAH inhibitors is based on extending the biological effects of endocannabinoids by increasing their levels in the peripheral nervous system (PNS) and central nervous system (CNS) [5,9,10]. Unlike classic neurotransmitters, endocannabinoids are not stored, but are synthesized locally “on demand” in response to physiological and pathological stimuli [11].

As demonstrated by earlier studies, CB1Rs are distributed throughout the CNS and PNS, mainly in many important sites of the pain pathway, such as periaqueductal central gray (PAG), rostral ventromedial medulla (RVM), and nucleus trigeminalis caudalis (NTC), as well as in the brain microvascular endothelial cells and astrocytes [12,13], while CB2R is expressed in the peripheral tissues, mainly in the cellular elements of the immune system, and in PAG and microglial cells in the brain. The activation of CBRs mainly causes antinociception, as well as plays an important role in maintaining the integrity of the blood–brain barrier (BBB) [10,12].

The cerebrospinal fluid (CSF) plays an important role in maintaining CNS homeostasis and ensuring optimal conditions for the functioning of neurons [14,15,16,17]. The transmission of signals between the nervous tissue and the CSF and blood occurs despite the preserved barriers in the brain, and its mechanism is still being studied [18]. There are several elements taking part in the regulation of the exchange between the blood, the brain, and the CSF. They include specialized ependymal tanycyte cells, which extend from the third ventricle to the perivascular space of the fenestrated capillary network [19,20], tanycyte-like cells in the periventricular organs [21], and the cerebrospinal fluid-contacting nucleus (CSF-contacting nucleus) located in the ventral PAG of the lower portion of the aqueduct (Aq) and the upper portion of the fourth ventricle (4 V) floor [22,23,24].

The CSF-contacting nucleus is believed to be a unique nucleus in the brain that participates in neuron–neuron and neuron–body fluid crosstalk, and thus possibly influences the regulation of multiple physiological processes [23,24,25,26]. Increasing evidence also suggests that the CSF-contacting nucleus mediates the transduction and regulation of pain signals and might be involved in the modulation of the descending inhibitory system [27,28,29]. However, the precise molecular mechanisms remain unclear.

Some of the endocannabinoids have been demonstrated to alter the permeability of the BBB in various pain disorders [16,30]. This may be due to their action in the periventricular area that is not protected by the BBB, or to the mediation of the function of the CSF-contacting nucleus [16,24,25,31]. We presumed that AEA and 2-AG synthesized in the brain tissue are released into the CSF and may act in a competitive way to regulate the function of the brain centers located in the vicinity of the cerebral ventricles and the fundus of the fourth ventricle, where the sensory and motor centers of the tested trigemino-hypoglossal reflex are located [32,33,34,35].

The aim of our study was to investigate the effect of the stimulation of the nociceptive/afferent terminals of the tooth pulp and treatment with the FAAH inhibitor URB597 on the release of endocannabinoids (AEA and 2-AG) into the CSF and to determine whether the activation of endogenous cannabinoid receptors (CB1 and CB2) in the mesencephalon, thalamus, and hypothalamus occurs during this treatment.

## 2. Materials and Methods

### 2.1. Animals and Anaesthesia

Adult male Long–Evans rats, weighing 330–350 g, of 3 months of age were randomly allocated to experimental groups in this study. The animals were housed under a constant temperature (22–24 °C) and maintained in sawdust-lined plastic cages under a 12 h light/dark cycle with free access to laboratory chow pellets and tap water ad libitum. The rats were anaesthetized with a single i.p. injection of chloralose solution at a dose of 150 mg·kg^−1^ body weight. In all of the experimental animals, the anesthesia persisted for the whole period of the experiment. For each experiment, groups of 6 animals were used. The animal sample sizes were determined based on our preliminary studies to ensure adequate statistical power. To minimize circadian influence, all experiments were performed between 8:00 a.m. and 12:00 a.m. after 7 days of acclimatization. The experimental protocol was approved by the Local Ethical Committee for Animal Experiments at the Medical University of Lodz (42/ŁB 103/2018; 42/ŁB 103-A-DLZ/2020; and 43/ŁB 103-B-NZP/2020) and was in accordance with the European Communities Council Directive 2010/63/EU. All experimental procedures were performed in accordance with the current guidelines for the care of laboratory animals (including the use of the 3 Rs procedures) and in accordance with the ARRIVE guidelines [36,37].

### 2.2. Chemicals

Control perfusion of the cerebral ventricles was conducted with artificial cerebrospinal fluid (aCSF), which was prepared according to Daniel and Lederis [38] and contained 120 mM NaCl, 4.8 mM KCl, 2.8 mM CaCl_2_, 1.2 mM KH_2_PO_4_, 1.3 mM MgSO_4_, 26 mM NaHCO_3_, 10 mM glucose, 1.0 g/L bovine serum albumin, and 0.1 g/L ascorbic acid (pH = 7.5). The solution was placed in a water bath at 37 °C and constantly gassed with carbogen (a mixture of 95% O_2_ and 5% CO_2_).

URB597 (Sigma Aldrich, Poznań, Poland), administered i.p., was dissolved in dimethyl sulfoxide (DMSO) and injected at a dose of 2 mg/kg 1 h before tooth pulp stimulation [9,39]. The solution was administered in 1 mL/kg volume [9].

### 2.3. Perfusion of Cerebral Ventricles in Rats

The perfusion of the cerebral ventricles and simultaneous recording of evoked tongue jerks (ETJ) in rats is a further modification of a method described previously [40].

**Scheme 1:**
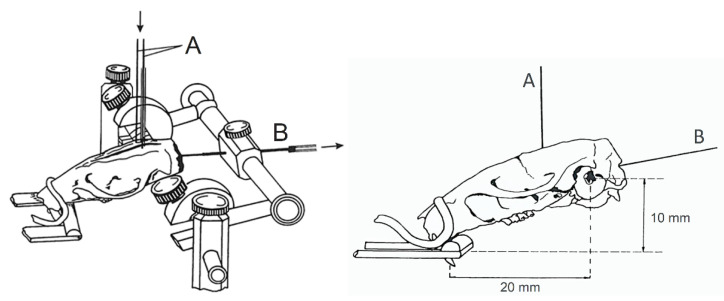
Position of a rat skull in a stereotaxic instrument adapted for the perfusion of cerebral ventricles. (**A**)—inflow cannula for the lateral ventricle. (**B**)—outflow cannula for the cerebello-medullary cistern. Description in the text.

The heads of the animals were immobilized in a simple stereotaxic instrument (Department of Precision Mechanics, Medical University of Lodz, Lodz, Poland) specially adapted for perfusion of cerebral ventricles in rats. The heads of the animals were immobilized by the introduction of ear bars into the external auditory meati and fixing the maxilla with an upper jaw clamp. The pallatum durum just behind the upper incisor was 10 mm below the intraauricular axis, so the animals noses were down. The interauricular axis was 60 mm above the surface of the instrument base on which the animals’ bodies were lain. This position of the animals’ heads in the stereotaxic instrument widened the space between the occipital bone of the cranium and the first cervical vertebrae (Scheme 1). 

**Scheme 2:**
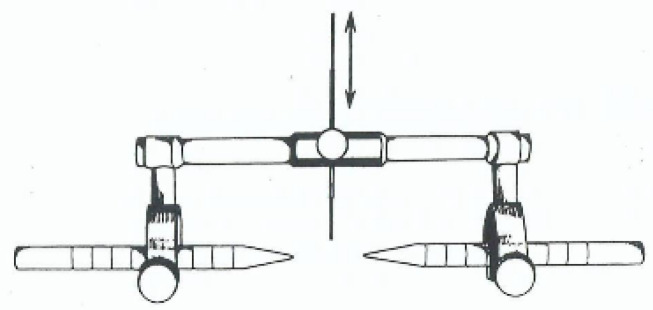
Holder of the cannula for the cerebello-medullary cistern. The cannula holder was rigidly attached to ear bars during the cerebral ventricle perfusion.

It allows the introduction of a cannula into the cerebello-medullary cistern and fixing it in a rigid position. The cannula for the cistern was composed of our guide stainless tube of 1.0 mm external diameter and an inner stainless steel tube of 0.6 mm external diameter. The outer tube with the inner tube inside was inserted through the skin and muscles to the atlanto-occipital membrane, which was punctured by the inner tube, the tip of which was introduced into the cistern. During the whole experiment, the cannula was rigidly fixed to the cannula holder attached to the ear bars (Scheme 2).

A fine polyethylene tube about 100 cm long filled with perfusion fluid was permanently connected to the cistern cannula. The free end of the polyethylene tube was kept about 60–80 cm below the animal’s head during cerebral ventricles perfusion. 

The skin of the animal’s head, anesthetized with 2% polocaine solution, was incised in the midline and the bones of the skull were exposed. On the basis of modified coordinates given by the Paxinos and Watson stereotaxic atlas [41], the sites for drilling holes to the lateral ventricles in the skull bones were determined. Using the stereotaxic instrument, two points were marked on the surface of the skull 6 mm anteriorly to the frontal interaural zero plane and 3 mm laterally on either side of the sagittal zero plane. Holes were drilled into the skull bones at the marked points. Two stainless-steel cannulae with an external diameter of 0.6 mm, connected through polyethylene tubes to a vessel with perfusion fluid and kept about 25 cm above the rat’s head, were inserted into the lateral ventricles through the drilled holes to a depth of 4 mm from the surface of the skull. Then, the vessel with perfusion fluid (aCSF solution) was lowered to obtain a perfusion rate ranging from 0.8 to 1.0 mL/20 min. 

### 2.4. Tooth Pulp Stimulation

After placing the animal’s head in a stereotaxic instrument, the tips of both lower incisors were cut off with a dental separator (Turbina NSK Pana-Max2 M-4, Tokyo, Japan) and stainless-steel wire electrodes (Endostar size 02–03, 25 mm, Poldent, Warsaw, Poland) were inserted into the pulp and fixed with dental cement (Duracryl Plus, SpofaDental, Inc, Prague, Czech Republic). The bipolar tooth pulp stimulation was delivered with a train of four electrical impulses of 200 Hz frequency, 3 ms single impulse duration, and 4–5 V amplitude using a programmed stimulator. Trains of 4 impulses were delivered to the pulp at 10 s intervals using a Grass stimulator (model S 4K, Medical Quincy, Waltham, MA, USA) coupled with a gate generator (RTX-310, OB-5 PEMIEC 439-1).

### 2.5. Recording Tongue Jerks

The tip of the animal’s tongue was attached with a silk thread to an isotonic rotating tensometric transducer (Department of Precision Mechanics, Medical University of Lodz, Poland). The amplitude of the electrical pulses was adjusted so that each pulse caused a distinct sharp backward movement of the stretched tongue to obtain a recorded amplitude of about 20 mm. Usually, this amplitude of ETJ was below one-fourth of the maximal amplitude evoked by supramaximal nerve stimulation. Throughout the whole experiment, the tongue was stretched with the same force of about 5.8 g by the lever of a strain gauge isotonic transducer, and the adjusted amplitude of the electric pulses was not changed. The amplitudes of ETJ by tooth pulp stimulation were recorded on paper using a Line Recorder TZ 4620 (Laboratorni Pristroje Prague, Prague, Czech Republic). The mean amplitude of ETJ by tooth pulp stimulation was regarded as an indicator of the magnitude of the trigemino-hypoglossal reflex.

### 2.6. Experimental Design

**Scheme 3:**
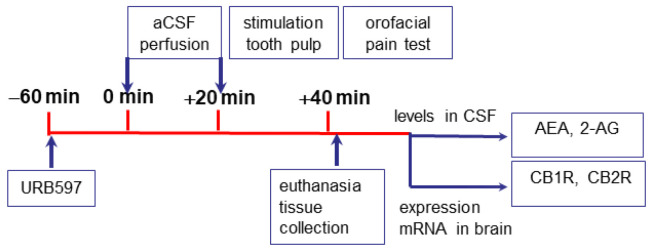
Experimental design. Description in the text.

CSF was collected from the cerebello-medullary cistern, and then 20 min perfusion of the lateral ventricles with artificial cerebrospinal fluid (aCSF) was conducted with the collection of perfusate portions from the cerebello-medullary cistern during electrical tooth pulp stimulation evoking the nociceptive tongue jerk reflex. The next group consisted of rats that received URB597. In this group, 20 min aCSF perfusion was conducted before tooth pulp stimulation for another 20 min (Scheme 3). The CSF for the molecular studies was frozen and stored at −70 °C for no longer than 2 weeks.

### 2.7. ELISA Test

The AEA and 2-AG concentrations were measured in rat CSF with commercial ELISA kits (MyBioSource, San Diego, CA, USA). The sensitivity of the tests was 0.1 ng/mL and 0.43 pg/mL for AEA and 2-AG, respectively. The protocols were performed according to the manufacturers’ instructions. A Stat-Matic Plate Washer II (Sigma-Aldrich) was used for the washing steps. The optical density was measured at 450 nm and the protein concentration of the samples was determined by interpolation from the standard curve; the intra-assay %CV was 1.42 for AEA and 5.73 for 2-AG.

Absorbance was read using a VICTOR^TM^ X4 Multifunctional Microplate Reader (Perkin Elmer, Waltham, MA, USA), and the results were analyzed with WorkOut 2.5 Software. The mean concentration of protein per mL was determined with reference to a four-parameter logistic curve (4-PL).

### 2.8. RNA Isolation and Gene Expression Analysis

#### 2.8.1. Total RNA Isolation

Total RNA isolation from the rat brain samples using a RNA extraction reagent, TRIZOL (Invitrogen Life Technologies), was performed according to the standard acid–guanidinium–phenol–chlorophorm method [42]. The absorbance of isolated RNA was measured using a spectrophotometer (model Pico200 Picodrop Microliter, Southern Labware, Cumming, GA, USA) at λ = 260 nm in order to determine the total RNA concentration. The isolated RNA was stored at −70 °C.

#### 2.8.2. Quality Analysis of Isolated RNA

The quality of the total RNA was checked with an Agilent RNA 6000 Nano Kit (Agilent Technologies, Santa Clara, CA, USA) in accordance with the manufacturer’s recommendations. An amount of 1 µL of RNA 6000 Nano dye was added to a test tube containing 65 µL of Agilent RNA 6000 Nano gel matrix and then centrifuged (10 min, 13,000× *g*). The gel–fluorescent dye mixture was applied to the surface of a Nano chip placed in a workstation. Then, 5 µL of RNA Nano marker was added to selected pits. Isolated samples of RNA and RNA size marker were subject to denaturation (2 min, 70 °C), and then 1 µL of the sample was pipetted into selected pits of the Nano chip and mixed (1 min, 2400 rpm). The quality of isolated RNA was checked using a 2100 Bioanalyzer (Agilent Technologies, Santa Clara, CA, USA). The level of degradation of the total RNA was determined with the use of an electrophoretogram and recorded RIN values. Only the samples with a RIN value of >7 were subject to further analysis.

#### 2.8.3. RT-PCR Reverse Transcription

A RT reaction was carried out using a TaqMan^®^ RNA Reverse Transcription Kit (Applied Biosystems, Waltham, MA, USA) based on the manufacturer’s recommendations, using Rn 01460701_g1, Rn 04342831_g1, and Rn 01775763_g1 probes specific for rat CB1R, CB2R, and GADPH cDNA, respectively (Applied Biosystems). The samples were incubated (30 min, 16 °C and 30 min, 42 °C) in a thermocycler (Biometra Tone, Analytik Jena GmbH, Jena, Germany). The reverse transcriptase was inactivated (5 min, 85 °C) and the obtained cDNA was stored at −20 °C.

#### 2.8.4. Real-Time PCR Reaction

A real-time PCR reaction was conducted using TaqMan^®^ Universal PCR Master Mix, No UNG (Applied Biosystems, Waltham, MA, USA), according to the protocol provided by the manufacturer. The reaction mixture ratio is presented in Table 1. To calculate the relative expression of miRNA genes, the Ct comparative method was used [43,44]. The level of rat CB1R and CB2R gene expression in rat brains was normalized in relation to the GADPH reference gene (Table 1).

Each target probe was amplified in a separate 96-well plate. All samples were incubated at 50 °C for 2 min and at 95 °C for 10 min, and then cycled at 95 °C for 30 s, at 60 °C for 30 s, and at 72 °C for 1 min; 40 cycles were performed in total. Standard curves were created for 2.5, 2.0, 1.5, 1.0, and 0.5 µL of the obtained cDNA and for each of the target genes. Fluorescence emission data were captured and the mRNA levels were quantified using the critical threshold (Ct) value. Analyses were performed with an ABI Prism 7000 Sequence Detection System (Applied Biosystems, Walham, MA, USA). Controls without RT and with no template cDNA were tested with each assay. The relative gene expression levels were obtained using the ∆∆Ct standard 2 ^−∆∆ct^ calculations and expressed as a fold change of the control sample [43,44]. Amplification-specific transcripts were further confirmed by obtaining the melting curve profiles.

### 2.9. Statistical Analysis

PRISM 9.2 (Graph-Pad Sotware Inc., La Jolla, CA, USA) and Statistica Version 13.1 (TIBCO, Palo Alto, CA, USA) were used to perform statistical analysis and generate plots. For the AEA and 2-AG levels, the statistical differences between groups were assessed using one-way ANOVA followed by Tukey’s multiple comparison post hoc test. Pearson correlations were used to assess the correlations between the AEA and 2-AG levels and the expression of CB1R and CB2R. All data are presented as the mean ± standard error of the mean (±SEM), and a *p*-value of <0.05 was considered statistically significant. The data and statistical analysis comply with the experimental design and analysis recommendations in pharmacology [45].

## 3. Results

### 3.1. Effect of Tooth Pulp Stimulation and URB597 Treatment on the AEA and 2-AG Concentration in the CSF

**Figure 1:**
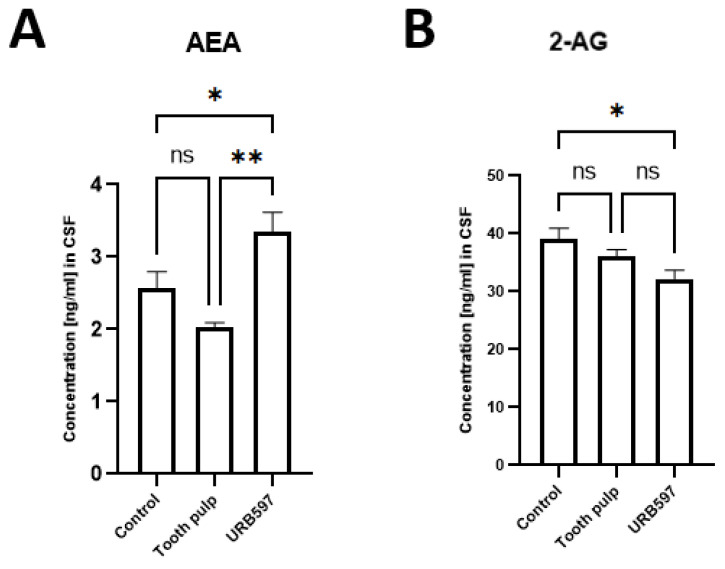
Comparison of the (**A**) AEA and (**B**) 2-AG concentrations in the CSF in controls and after tooth pulp and URB597 treatment. The data represent the mean ± SEM of 6 rats per group. Statistical significance was assessed using one-way ANOVA and a post hoc multiple-comparison Tukey test. Asterisks denote the significance level (* *p* < 0.05, ** *p* < 0.01, ns—not statistically significant).

ANOVA showed that nociceptive stimulation and antinociceptive treatment induced changes in the AEA concentration (F_2, 15_ = 10.37, *p* = 0.002). Peripheral treatment with a FAAH inhibitor, URB597, caused a significant increase in the AEA concentration in the rat CSF compared with the controls (*p* = 0.042) and rats treated with tooth pulp stimulation (*p* = 0.001). Tooth pulp stimulation did not change the AEA significantly in comparison with the controls (*p* = 0.190; Figure 1A).

Similarly, differences were found in the 2-AG concentration between groups (F_2, 15_= 5.344, *p* = 0.018). In particular, antinociceptive treatment with URB597 caused a significant decrease in the 2-AG concentration (*p* = 0.014). Tooth pulp stimulation had no influence on the 2-AG concentration in the rat CSF (*p* = 0.3330), Figure 1B.

### 3.2. Correlations between AEA and 2-AG Concentrations in CSF and CB1R and CB2R Expression in the Rat Brain Structures

**Figure 2:**
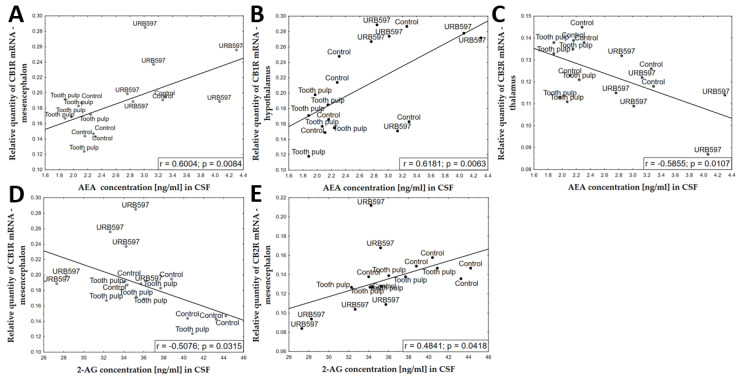
Statistically significant correlations between the concentrations of endocannabinoids in the CSF and cannabinoid receptor expression in the brain structures of rats: (**A**) correlation between AEA and CB1R expression in the mesencephalon, (**B**) correlation between AEA and CB1R expression in the hypothalamus, (**C**) correlation between AEA and CB2R expression in the thalamus, (**D**) correlation between 2-AG and CB2R expression in the mesencephalon, and (**E**) correlation between 2-AG and CB2R expression in the mesencephalon.

The correlations between the concentration of endocannabinoids and the expression of cannabinoid receptors in the mesencephalon, thalamus, and hypothalamus were evaluated. Table 2 summarizes the correlation coefficients and *p*-values of all examined correlations. We observed statistically significant correlations between the AEA concentration in the CSF and the CB1R mRNA expression in the mesencephalon (Pearson correlation, r = 0.60, *p* = 0.008) (Figure 2A) and hypothalamus (r = 0.62, *p* = 0.006) (Figure 2B). Conversely, a moderate negative correlation was observed between AEA and CB2R expression in the thalamus (r = −0.59, *p* = 0.011) (Figure 2C). 

Among the associations between 2-AG and cannabinoid receptor expression in the brain structures, we found a moderate negative correlation with CB1R mRNA expression in the mesencephalon (r = −0.51, *p* = 0.032) (Figure 2D). Furthermore, a moderate positive correlation between 2-AG and CB2R in the mesencephalon was observed (r = 0.48, *p* = 0.042) (Figure 2E). 

As the analyzed variables had normal distributions, the Pearson correlation was selected for analysis. Acknowledging that it may not be an ideal method to assess relationships between biological processes, which would not be linear in a larger sample size, we calculated Spearman correlations. The results obtained by both methods were similar; the same pairs of variables maintained their significance (Supplementary Table S1). 

## 4. Discussion

Previous studies have shown that the CSF plays an important role in maintaining CNS homeostasis and the BBB maintains an environment that allows the neurons to function properly [14,15,46]. It is a selectively permeable barrier that regulates the transport of metabolites to and from the CNS [47]. The triggering mechanism of BBB disturbances in orofacial pain is not fully understood.

Low levels of the two main endocannabinoids have been correlated with headaches [10,48,49,50,51]. Some endocannabinoids have been shown to alter its permeability in migraines and other headaches [52,53], and the cannabinoid receptors (CBRs) play an important role in maintaining the BBB’s integrity by restoring the tightness of the tight junctions [12,30,47].

To understand the role of endocannabinoids in orofacial pain better, we examined the effect of tooth pulp stimulation and treatment with URB597 on their release into the CSF, and we assessed the correlations between the eCB concentrations and the expression of CBRs in the mesencephalon, thalamus, and hypothalamus, i.e., in the regions that are involved in the transmission and modulation of pain signals.

We observed that nociceptive stimulation and antinociceptive treatment induced changes in the AEA and 2-AG concentrations. Peripheral treatment with the FAAH inhibitor URB597 caused a significant increase in the rats’ AEA concentration in the CSF compared with the controls and rats subjected to tooth pulp stimulation (Figure 1A), and resulted in a significant decrease in the 2-AG concentration in CSF (Figure 1B). In contrast, tooth pulp stimulation did not change the AEA and 2-AG concentrations significantly versus the controls (Figure 1A,B).

Our results show that AEA and 2-AG act differently because they have different regulatory mechanisms, which are related to their distribution in the CNS fibers containing AEA that extend widely throughout the brain and reach the PAG and RVM, but they mainly project along the ventricles of the brain and are in close contact with the lining of the third and fourth ventricles and with neurons in contact with the CSF [19,20,21,22,23,24,31]. Thus, the active transport of AEA to the CSF could take place via the specialized cells of the tanycyte lining extending from the third ventricle of the brain and via the CSF-contacting nucleus [54]. Some AEA may have also entered the CSF via the circumventricular organs (CVOs), via the Virchow–Robbin perivascular spaces, and via the BBB [55,56,57]. It is known that CSF is in constant exchange with the extracellular fluid (ECF), which means that the endogenous cannabinoids present in the CSF compartment may migrate from their site of release [58].

**Scheme 4:**
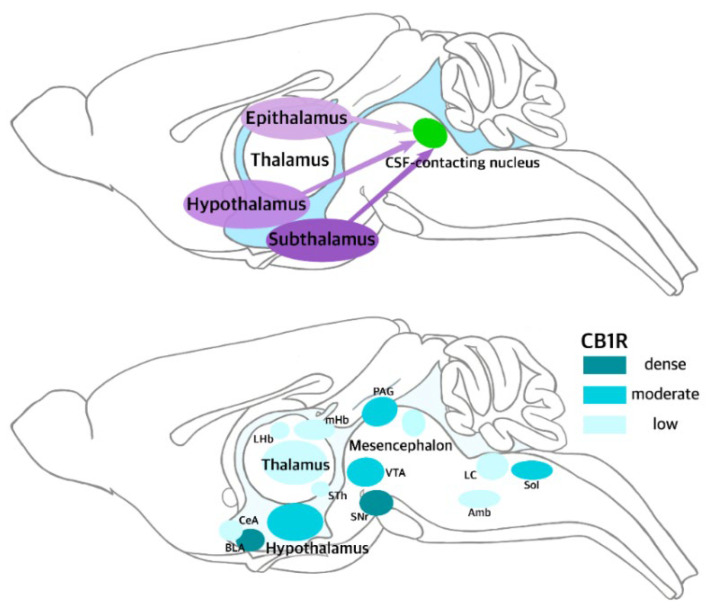
The upper schematic diagram shows projections of the connections of the mesencephalon, thalamus, and hypothalamus (epithalamus and subthalamus) to the cerebrospinal fluid-contacting nucleus (CSF-contacting nucleus). The lower diagram shows the distribution of CB1Rs in these areas of the diencephalon and brainstem. The distribution of CB1Rs is indicated by the circular shapes with low (pale turquoise), moderate (light turquoise), and high (dark turquoise) expression. Data for figures were taken and adapted from Song (© 2020 Song, Li, Zhai, Li, Bao Shan, Hong Cao and Zhang) ([25] and Befort (©Befort) [61] under the terms of the Creative Commons Attribution License (CC BY)).

The unique features of the morphological connections of the CSF-contacting nucleus imply that it is the only area within the CNS involved in bidirectional signal transduction and the transport of bioactive substances between the brain parenchyma and the CSF [27,59,60]. This nucleus receives extensive projections from the hypothalamus, epithalamus, and subthalamus, i.e., the brain structures important for the generation and propagation of pain signals specific to orofacial pain (Scheme 4) [25].

Routes containing AEA that mainly lead from the hypothalamus have been found to extend to destinations outside the hypothalamus [28,62]. Based on these connections and the distribution of CB1Rs, we suggest that the CSF-contacting nucleus mediated AEA transmission to the CSF and the transmission of pain signals, and may be involved in the regulation of the descending pain inhibitory system [25,31,63,64,65].

Increasing endocannabinoids by inhibiting FAAH has the benefit of activating CB1Rs in the nociceptive pathways with high endocannabinoid turnover [7,10]. The systemic administration of the FAAH inhibitor URB597 has been found to lead to the accumulation of AEA in the brain and its release to the CSF without affecting the 2-AG levels [66], which is in line with our studies. In our experimental model, 2-AG probably does not affect the BBB’s permeability. As it follows from the research of other authors, 2-AG can activate receptors that modify the BBB’s permeability (i.e., CB2R and PPARα), so it is unclear why their effects are dissimilar to those of AEA in our experimental model [16,67]. However, it is known that endocannabinoids have a complex pharmacology that can be explained by many phenomena, including the transport mechanisms, allosteric modulation, and activation of receptors other than CB2Rs. It may be difficult to activate CB2Rs without affecting other receptors, or a stronger noxious stimulus is needed to activate 2-AG. The lack of effect of the stimulation of the trigeminal nerve sensory branches on the 2-AG content in the CSF in our study may also be explained by the fact that pain stimulation of the tooth pulp causes the release of significant amounts of 2-AG from dendrites into the ECF without increasing the concentration of 2-AG in the CSF. The above does not necessarily mean that the 2-AG released into the ECF does not reach the cerebral ventricles, but rather, it suggests that many of the mediator molecules are lost on their long-term passage into the ventricles, as it binds to various receptors and is broken down by enzymes, or that tooth pulp stimulation cannot depolarize enough nerve fiber endings extending close to the lining, possibly due to too little 2-AG release from the brain and the varying proportion of CB2Rs in the brain.

**Scheme 5:**
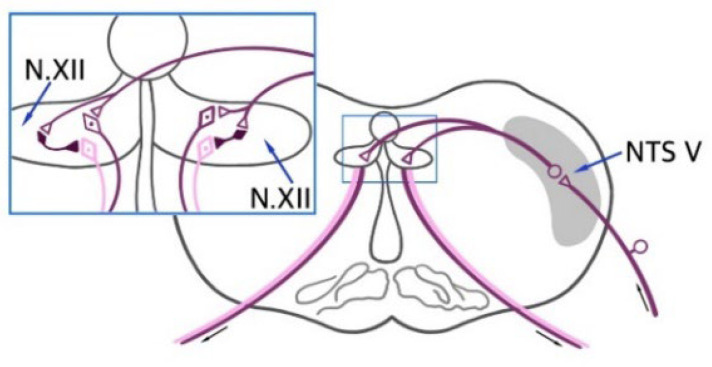
A scheme of the trigemino-hypoglossal reflex arc. In trigemino-hypoglossal reflexes, such as tongue jerks (ETJ) evoked by tooth pulp stimulation, the impulsation from n.V sensory nuclei was transferred to the ipsi- and contralateral hypoglossal nerve nucleus, where it stimulated the motoneuron of the tongue-withdrawing muscles, and the excitation of the inhibitory interneuron caused the inhibition of the tongue-protrusion-muscles motoneuron. NTS V—n.V spinal tract nucleus; N.XII—hypoglossal nerve nucleus; light-colored triangle—the excitatory synapse, dark triangle—the inhibitory synapse.

This different effect of URB597 treatment and tooth pulp stimulation on the concentration of AEA and 2-AG in the CSF can also be explained by their different effects on the neurons of the sensory nuclei of the trigeminal nerve, intermediary neurons, n.XII motor neurons, and structures modulating conduction through the elements of the trigeminal-hypoglossal reflex. The trigemino-hypoglossal reflex arc extends below the floor of the IV ventricle between the sensory nucleus of the trigeminal nerve and the motor nucleus of the hypoglossal nerve (Scheme 5).

The biological effects of endocannabinoids in humans and animals are mainly related to the distribution of their specific receptors in the CNS [4]. CB1Rs and CB2Rs play an important role in maintaining the integrity of the BBB because they are present on the brain’s microvascular endothelial cells and astrocytes [30]. Therefore, in the next step, the correlations between the concentration of endocannabinoids and expression of cannabinoid receptors in the mesencephalon, thalamus, and hypothalamus were evaluated. We observed a statistically significant high correlation between the AEA concentration in the CSF and CB1R mRNA expression in the mesencephalon (Figure 2A) and hypothalamus (Figure 2B), and a moderate positive correlation between 2-AG and CB2R mRNA in the mesencephalon (Figure 2E). On the other hand, we observed a moderate negative correlation between AEA and CB2R mRNA expression in the thalamus (Figure 2C), and between 2-AG and CB1R mRNA expression in the mesencephalon (Figure 2D). The above results allow us to conclude that the inhibition of FAAH activity by URB597 resulted in an increase in the concentrations of AEA and CB1R mRNA and a decrease in the expression of CB2Rs. These observations suggest that CB1Rs were activated by the increased levels of AEA in the investigated brain structures produced by the administration of URB597, and that the analgesic effect of URB597 requires the CB1R in response to orofacial pain. On the other hand, URB597 had no significant effect on the concentration of 2-AG in the CSF. The negative effect of 2-AG on the BBB’s permeability in orofacial pain may be manifested through the greater activation of receptors other than CB. Interestingly, 2-AG was less effective in our experiments. despite the fact that it is present in the brain in higher concentrations than AEA and can stimulate equally through CB1Rs and CB2Rs [68]. It may potentially be due to lower levels of 2-AG access to the presynaptic CB1Rs, as well as to the fact that the 2-AG-degrading enzyme is closer to the CB1R target than AEA. Another potential explanation is that AEA and 2-AG have different anatomical distributions of their FAAH-degrading enzymes, generally localized at postsynaptic sites, whereas, for MAGL, they are localized in presynaptic terminals [8,69]. Furthermore, our findings are consistent with the concept that AEA and 2-AG can independently regulate pain perception, as they have different regulatory mechanisms specific to the experimental model, sex, and the used animal species [70,71,72]. Cannabinoid agonists have been reported to bind with greater affinity to CB1Rs in female than male rats, probably contributing to the greater antinociceptive effects observed in females compared with male rats [72,73]. It has also been described that there are behavioral differences between various rat strains following the intake of cannabinoids [74]. 

Our results are consistent with the role of AEA-mediated endocannabinoid signaling in migraines and suggest that a FAAH inhibitor may offer a new therapeutic option for the prevention of orofacial pain. Further experimental and clinical studies are still needed to confirm the validity of our speculations.

In summary, we are the first to report information on the effect of a FAAH inhibitor and tooth pulp stimulation on the endocannabinoid concentrations in the CSF along with the correlations between cannabinoid receptors in the brain and orofacial pain in rats. In animal models of orofacial pain, endocannabinoid tone modulation via the inhibition of endocannabinoid-catabolizing enzymes has been an area of particular interest of research. We determined that AEA, but not 2-AG, is significantly elevated in the CSF after treatment with a FAAH inhibitor, while tooth pulp stimulation had no effect on the AEA and 2-AG levels in the CSF. We found positive correlations between the CSF AEA concentration and CB1R expression in the mesencephalon and hypothalamus, as well as between 2-AG in the CSF and mesencephalic CB2R, and negative correlations between the CSF level of AEA and thalamic CB2R expression, and between the CSF 2-AG concentration and CB1R expression in the midbrain. As it follows from the above, the two major endocannabinoids may be differentially involved in orofacial pain. In addition, our findings may be consistent with the emerging concept that AEA and 2-AG have different regulatory mechanisms, and that AEA is preferentially involved in pathological events. The lack of research on the endocannabinoid system in orofacial pain is a clear gap in the literature and, therefore, there is an urgent need for experimental research in this field.

### Limitations

The following limitations of the study should be considered: first, the sample size was relatively small. A significant limitation of this study was also the lack of correlation of the obtained results between AEA and CB2R and between 2-AG and CB1R. Therefore, additional studies are needed to determine if the endocannabinoid tone is decreased in orofacial pain due to a reduced number or reduced sensitivity of the CB1/CB2 receptors, or the overactivity of the FAAH and MAGL degradation enzyme (for AEA and 2-AG, respectively).

## 5. Conclusions

Our study shows that the FAAH inhibitor URB597 causes a significant increase in the AEA concentration and a significant decrease in the 2-AG concentration in the CSF and produces CB1R-mediated antinociception in orofacial pain in rats. Our observations provide evidence for the involvement of the endocannabinoid system in orofacial pain. The modulation of this system through the peripheral inhibition of FAAH may be a promising therapeutic approach to the treatment of orofacial pain.

## Figures and Tables

**Table 1:** PCR primer sequences used for the RT-PCR analysis of mRNA.

Gene	Forward Primers	Reverse Primers
*GAPDH*	5′-GTCGCTGTTGAAGTCAGAGGAG-3′	5′-CGTGTCAGTGGTGGACCTGAC-3′
*CB1R*	5′-CTACTGGTGCTGTGTGTCATC-3′	5′-GCTGTCTTTACGGTGGAATAC-3′
*CB2R*	5′-AGGTTGCATTCCCAACAGAC-3′	5′-TTAGTTCCTCTGGGCAATGG-3′

**Table 2:** Pearson correlation coefficients and associated *p*-values for endocannabinoids (AEA/ 2-AG) and the expression of CB1R/CB2R. Blue indicates negative R values and red indicates positive R values.

Endocannabinoids	Relative Quantity of CB1R mRNA—Mesencephalon	Relative Quantity of CB1R mRNA—Thalamus	Relative Quantity of CB1R mRNA—Hypothalamus	Relative Quantity of CB2R mRNA—Mesencephalon	Relative Quantity of CB2R mRNA—Thalamus	Relative Quantity of CB2R mRNA—Hypothalamus
AEA CSF [ng/mL]	0.60 *p* = 0.008	0.26 *p* = 0.292	0.62 *p* = 0.006	−0.14 *p* = 0.587	−0.59 *p* = 0.011	−0.21 *p* = 0.398
2-AG CSF [ng/mL]	−0.51 *p* = 0.032	−0.41 *p* = 0.093	−0.28 *p* = 0.267	0.48 *p* = 0.042	0.32 *p* = 0.193	0.40 *p* = 0.104

## Data Availability

The data presented in this study will be made available upon reasonable request.

## Supplementary Materials

The following supporting information can be downloaded at: https://www.mdpi.com/article/10.3390/cimb44050164/s1.

## Abbreviations

2-AG	2-arachidonoylglycerol
aCSF	artificial cerebrospinal fluid
AEA	N-arachidonyl ethanol amine, anandamide
BBB	blood–brain barrier
CB	cannabinoid
CB1R	cannabinoid receptor type 1
CB2R	cannabinoid receptor type 2
CBR	cannabinoid receptor
CNS	central nervous system
CSF	cerebrospinal fluid
DAGL	diacylglycerol lipase
DMSO	dimethyl sulfoxide
EC	endocannabinoid
ECS	endocannabinoid system
ETJ	evoked tongue jerks
FAAH	fatty acid amide hydrolase
MAGL	monoacylglycerol lipase
NTC	nucleus trigeminalis caudalis
PAG	periaqueductal central gray
RVM	rostral ventromedial medulla
URB597	FAAH inhibitor-degrading enzyme of AEA
