# Supplementary material for: Release of Endocannabinoids into the Cerebrospinal Fluid during the Induction of the Trigemino-Hypoglossal Reflex in Rats
Source: Curr Issues Mol Biol. 2022 May 23;44(5):2401–16. doi: 10.3390/cimb44050164 (PMC9164053; doi:10.3390/cimb44050164)
Supplement: Supplementary file 1 [file cimb-44-00164-s001.zip › cimb-1716905-supplementary.pdf]

Table S1. Spearman Correlation Coefficients and associated p-values for endocannabinoids (AEA/2-AG) and expression of CB1R/CB2R.

Pairs of variables	Spearman R	t(N-2)	p
**Relative quantity of CB1R mRNA - mesencephalon** & AEA concentration [ng/ml] in CSF	0.5661	2.7471	0.0143
**Relative quantity of CB1R mRNA - mesencephalon** & 2-AG concentration [ng/ml] in CSF	-0.6040	-3.0316	0.0079
**Relative quantity of CB1R mRNA - thalamus** & AEA concentration [ng/ml] in CSF	0.3696	1.5913	0.1311
**Relative quantity of CB1R mRNA - thalamus** & 2-AG concentration [ng/ml] in CSF	-0.3044	-1.2784	0.2193
**Relative quantity of CB1R mRNA - hypothalamus** & AEA concentration [ng/ml] in CSF	0.5658	2.7450	0.0144
**Relative quantity of CB1R mRNA - hypothalamus** & 2-AG concentration [ng/ml] in CSF	-0.2095	-0.8570	0.4041
**Relative quantity of CB2R mRNA - mesencephalon** & AEA concentration [ng/ml] in CSF	-0.0073	-0.0290	0.9772
**Relative quantity of CB2R mRNA - mesencephalon** & 2-AG concentration [ng/ml] in CSF	0.6056	3.0440	0.0077
**Relative quantity of CB2R mRNA - thalamus** & AEA concentration [ng/ml] in CSF	-0.3673	-1.5794	0.1338
**Relative quantity of CB2R mRNA - thalamus** & 2-AG concentration [ng/ml] in CSF	0.2272	0.9330	0.3647
**Relative quantity of CB2R mRNA - hypothalamus** & AEA concentration [ng/ml] in CSF	-0.1829	-0.7444	0.4675
**Relative quantity of CB2R mRNA - hypothalamus** & 2-AG concentration [ng/ml] in CSF	0.2676	1.1108	0.2831
